# The effects of probiotic *Saccharomyces boulardii* on the mental health, quality of life, fatigue, pain, and indices of inflammation and oxidative stress in patients with multiple sclerosis: study protocol for a double-blind randomized controlled clinical trial

**DOI:** 10.1186/s13063-019-3454-9

**Published:** 2019-06-24

**Authors:** Dawood Aghamohammadi, Hormoz Ayromlou, Neda Dolatkhah, Fatemeh Jahanjoo, Seyed Kazem Shakouri

**Affiliations:** 1 Department of Anesthesiology, Faculty of Medicine, Tabriz University of Medical Sciences, Tabriz, Iran; 2 Neuroscience Research Center, Aging Research Institute, Tabriz University of Medical Sciences, Tabriz, Iran; 3 Physical Medicine and Rehabilitation Research Center, Aging Research Institute, Tabriz University of Medical Sciences, Tabriz, Iran

**Keywords:** Multiple sclerosis, Probiotics, *Saccharomyces boulardii*, Inflammation, Oxidative stress

## Abstract

**Background:**

The relationship between gut dysbiosis and inflammatory diseases including multiple sclerosis (MS) is presently recognized as an important health issue. It has been established that some bacterial probiotic strains are effective in treating MS. This study will investigate the effect of yeast probiotic *Saccharomyces boulardii* (SB) supplements on mental health, quality of life, fatigue, pain, and indices of inflammation and oxidative stress in MS patients.

**Methods/design:**

In this double-blind randomized controlled two-group parallel trial, 50 MS patients who meet the inclusion criteria will be recruited from outpatient settings. They will be randomly allocated to 4 months of daily placebo or the SB probiotic intervention. Blood samples will be taken from each participant at the baseline and after the intervention period to assess inflammation and oxidative stress. The primary endpoint will be the changes in their mental health evaluated by the 28-item General Health Questionnaire. The secondary endpoints include changes in: (1) quality of life, evaluated by the 36-item Short Form Questionnaire, (2) fatigue, evaluated by the Fatigue Severity Scale, (3) pain, evaluated by a visual analogue scale, and (4) serum levels of indices of inflammatory stress (high-sensitivity C-reactive protein) and oxidative stress (malondialdehyde and total antioxidant capacity). Moreover, any adverse events and side effects due to the intervention will be documented.

**Discussion:**

There is a need to discover safe and practical methods for managing the symptoms of MS. This trial will gather evidence on the effects of a probiotic.

**Trial registration:**

Iranian Clinical Trial Registry, IRCT20161022030424N1. Registered on 9 April 2018.

**Electronic supplementary material:**

The online version of this article (10.1186/s13063-019-3454-9) contains supplementary material, which is available to authorized users.

## Background

Multiple sclerosis (MS) is a neurodegenerative immune-mediated inflammatory disease distinguished by damage to the myelin sheaths of axons in the central nervous system [1]. MS affects at least 2.3 million people globally and is among the most frequent causes of neurological disabilities in young adults, particularly women [2].

Evidence linking diet to inflammation has emerged in recent years [3, 4]. This may be important for MS, since damage due to inflammation is the main stimulus for the neural damage seen in MS. There is an enhanced release of pro-inflammatory cytokines in MS, resulting in severe peripheral and central inflammation and leading to myelin demolition and axon destruction [5]. Previous studies have also confirmed that oxidative stress plays a fundamental role in MS pathophysiology [6, 7]. On the other hand, the gut microbiota is significantly affected by diet. Various scientific studies have attempted to evaluate the influence of gut microbiota and diet on the symptoms of MS.

In recent years, many animal and preclinical studies have established that the gut microbiota plays an important role in various aspects of human physiology, such as immunity, and it can regulate brain signals through the “microbiome–gut–brain axis” [8, 9]. Studies of experimental autoimmune encephalomyelitis, the mouse model of MS, have also shown that a disturbance to the gut microbiota affects the vulnerability to autoimmune encephalomyelitis [10, 11]. Moreover, variations in the gut microbiome of MS patients and dysbiosis have been suggested by a number of studies as possible factors [12–14].

If we accept that the gut microbiota affects the symptoms of chronic diseases such as MS, then we may be able to manage these diseases through changing the composition of the gut microbiota by adopting the right lifestyle or via probiotics. The effect of probiotic supplements in MS patients has been shown in a number of studies. Tamtaji et al. [15] showed that taking daily probiotic supplements containing *Lactobacillus acidophilus*, *L. casei*, *Bifidobacterium bifidum*, and *L. fermentum* (2 × 10^9^ colony-forming units (CFU) per g each) for 12 weeks down-regulates the expression of the messenger RNA of inflammatory markers of interleukin-8 and tumor necrosis factor alpha in mononuclear cells from the peripheral blood of MS patients. These probiotic supplements also improve Expanded Disability Status Scale scores and mental health, as measured with the Beck Depression Inventory, the 28-item General Health Questionnaire (GHQ-28), and the Depression Anxiety and Stress Scale [16]. According to Tankou et al. [17], probiotic supplements lead to an anti-inflammatory immune reaction, in the form of a reduced number of inflammatory monocytes. They may have a synergistic effect with new MS medications.

Heretofore, researchers often selected strains of bacterial species naturally present in the intestinal flora, such as lactobacilli and bifidobacteria. Thus, there is limited data on the effects of *Saccharomyces boulardii* (SB) supplements in MS patients. SB, which is a probiotic yeast, has unrivaled physiological attributes. It is stable under different pH levels, temperatures and when exposed to bile salts and gastrointestinal enzymes [18]. Numerous clinical investigations have shown that SB is better than other probiotics such as *Lactobacillus* and *Bifidobacterium* [19–22] in reestablishing the gut microbiota and aiding digestion [23, 24]. Moreover, it is resistant to antibiotics because it is a yeast [25].

On the other hand, recently published papers have recommended further studies on SB [26]. Thus, this trial will study the effects of SB supplements on mental health, quality of life, fatigue, pain, and indices of inflammation and oxidative stress.

## Method/design

### Study design

**Fig. 1:**
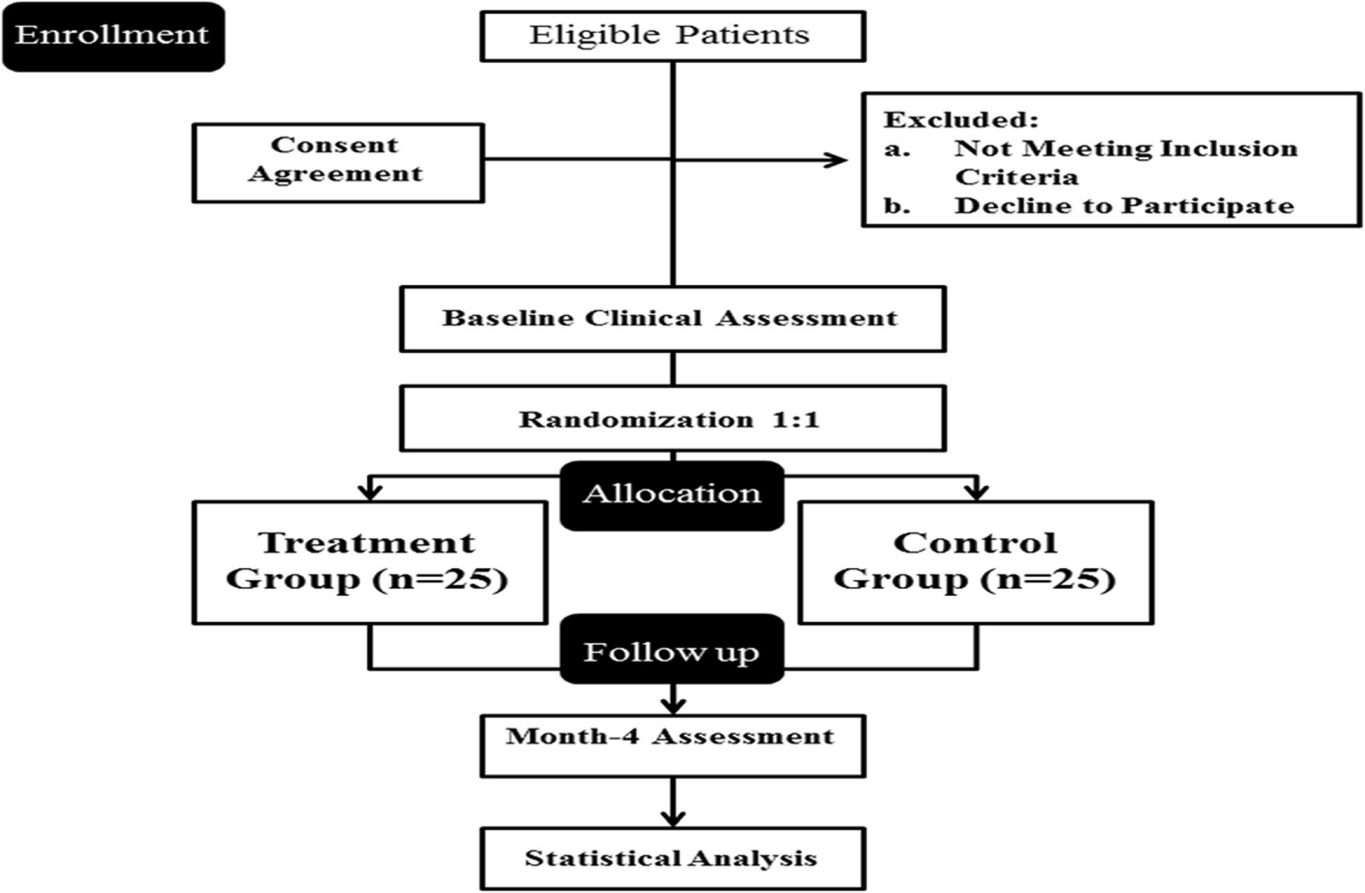
Participant flow diagram

The present study is a prospective randomized double-blind placebo-controlled parallel-group clinical trial to evaluate whether probiotic SB treatment for 4 months can improve mental health, quality of life, fatigue, pain, and indices of inflammation and oxidative stress in patients with MS. The researchers and the subjects are blind to the group assignment since the probiotic supplements and the placebo are completely identical and indistinguishable from one another. Enrollment began in June 2018 (Fig. 1).

### Hypothesis

Compared with a placebo, probiotic supplements containing SB (10^10^ CFU per capsule) will improve mental health, quality of life, fatigue, pain, and serum levels of indices of inflammatory and oxidative stress in MS patients.

### Specific objective

This study aims to determine the effects of probiotic SB supplements on the mental health, quality of life, fatigue, pain, and serum levels of indices of inflammatory and oxidative stress in MS patients.

### Subjects

#### Allocation

Patients attending outpatient clinics at the Emam Reza Hospital of Tabriz University of Medical Sciences who have been diagnosed with relapsing–remitting MS will be sent information about the project, including its methods and objectives. They will then be invited to an initial assessment to determine if they meet the inclusion and exclusion criteria. The protocol and trial procedures will be described to all participants. They will have an opportunity to discuss any issues, so that they are fully informed about all trial procedures. After informed consent has been obtained by the principal investigator, the patients will be randomly assigned to receive either the probiotic supplement or the placebo for 4 months.

#### Inclusion criteria

Patients can be included if they meet all the inclusion criteria:

- They are aged 18–55 years.
- They have clinically definite relapsing–remitting MS diagnosed according to the McDonald criteria.
- They have an Expanded Disability Status Scale score ≥4.5.
- They have been referred to an outpatient clinic of the Emam Reza Hospital of Tabriz University of Medical Sciences.
- They are able to answer verbal questions.

#### Exclusion criteria

Patients will be excluded if they meet any of the exclusion criteria:

- They are in an acute or severe phase of MS.
- At the beginning of the study, they are undergoing an active relapse.
- They smoke or consume alcohol.
- They are pregnant.
- They have used antibiotics or probiotics within the past 3 months.
- They have used corticosteroids within the past month.
- They have acute gastrointestinal problems within a month before or during the trial.
- They have been treated with interferon within 30 days prior to study enrolment.
- They have taken teriflunomide, fingolimod, or dimethyl fumarate within the past 3 months, natalizumab within the past 6 months, or rituximab, cyclophosphamide, or other immunosuppressive drugs within the past year.

#### Sample size

The main objective of this study is to determine the effect of probiotic SB supplements in comparison with a placebo on the mental health of MS patients, as evaluated by the GHQ-28. A similar study [16] found standard deviations of 6.2 and 6.4 in GHQ-28 scores for the intervention and placebo groups, respectively, and 6.5 was considered as the minimal clinically important difference. Based on a type 1 error rate (alpha) of 0.05 and a type 2 error rate (beta) of 0.10 (power = 90%) and using a two-way test, 20 participants are required in each group. Since we expect five dropouts per each group, the sample size is 25 patients per group.

#### Randomization and blinding

Random Allocation Software [27] was used to generate block-randomized lists. Altogether, 50 eligible participants will be randomized by a trained individual. The randomization ratio will be 1:1. We will stratify the randomization based on three variables, which will be equally distributed across the two groups: (1) body mass index, (2) age, and (3) type of medication. The random number list is stored securely at the Physical Medicine and Rehabilitation Research Center. The researchers do not have access to the list. To maintain the blinding, the allocation will be kept in opaque envelopes numbered consecutively from 1 to 100. None of the participants, the treating clinicians, or the outcome assessors will be aware of the intervention group status.

#### Lifestyle guidance

Participants in both groups will be given nutritional and lifestyle recommendations by a dietitian, which emphasize: (1) increasing their consumption of fruit and vegetables, (2) decreasing their consumption of energy-dense and low-nutrient density foods and drinks, (3) potion size management, and (4) increasing physical activity.

#### Intervention group

The intervention group will take BioDigest capsules once daily for 4 months. Each capsule contains 250 mg of SB (10^10^ CFU) plus a lactose filler and a magnesium stearate lubricant. The intervention capsules will be produced and packed by Takgene Pharmaceutical Company, Tehran, Iran.

#### Control group

The control group will take placebo capsules once daily for 4 months. In terms of shape, size, taste, smell, and other exfoliation characteristics, they are quite similar to the Biodigest capsules except that they do not contain any microorganisms. The placebo capsules will also be produced and packed by Takgene Pharmaceutical Company, Tehran, Iran.

### Outcomes

#### Participant timeline

**Fig. 2:**
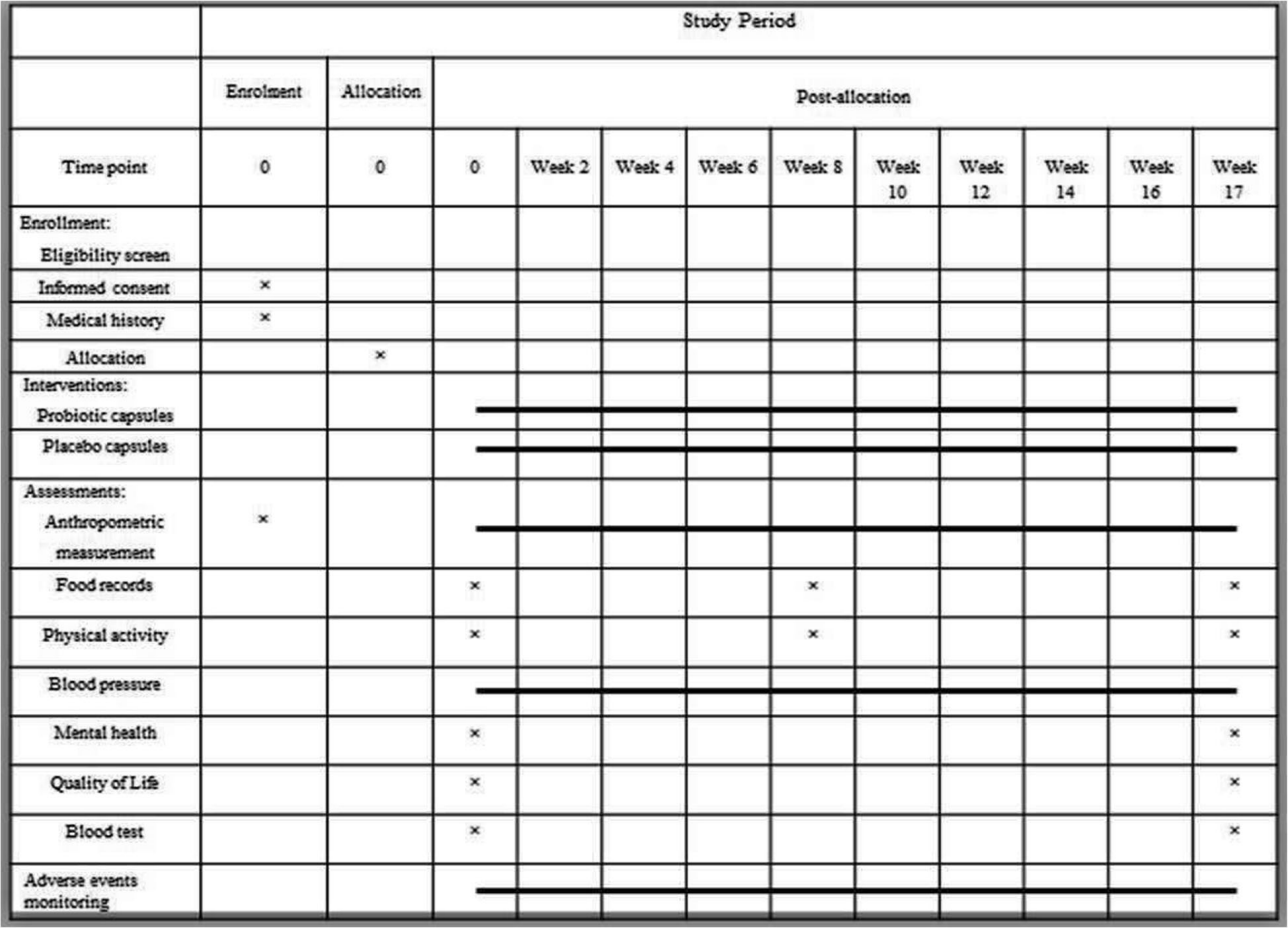
SPIRIT schedule of enrolment, interventions, and assessments

Figure 2 is the timetable of follow-up visits and measurements. It follows the SPIRIT guidelines. Each participant will visit the study center every 2 weeks. During these follow-up visits, they will receive sufficient capsules for the next 14 days. Throughout the study, participants will be contacted weekly by telephone to monitor their intake of the supplements and to prevent dropouts. If any participant discontinues with the trial, follow-up data will still be collected for them.

#### Primary outcome

The primary outcome is the difference in mental health evaluated by the GHQ-28 [28] in MS patients receiving probiotic supplements and the placebo.

#### Secondary outcomes

All physicians and researchers involved in this study will attend a workshop before the first participant is recruited to ensure that the assessments and data collection are conducted properly. In this study, the following data will be collected:

1. Checklist: This covers the inclusion criteria. It is completed at the baseline and indicates whether the patient is eligible for participation.
2. Demographic questionnaire: This includes age, level of education, employment status, income, age of first menstrual period, etc.
3. Mental health (Section 2.5.6)
4. Quality of life (Section 2.5.7)
5. Fatigue Severity Scale: This nine-item tool is used to evaluate fatigue for different chronic conditions and disorders including MS [29]. The applicability of the Persian version of the questionnaire to assess fatigue in MS patients has been established [30].
6. Visual analogue scale: This is a simple and commonly used instrument for measuring changes in the severity of pain [31].
7. Weight
8. Food intake (Section 2.5.9)
9. Physical activity (Section 2.5.5).
10. Blood pressure
11. Blood tests (Section 2.5.8)

#### Compliance

The participants are asked to bring their unused capsules to each of the fortnightly follow-ups. They are also asked to keep a daily checklist of their intake of the supplements. Participants will be considered as non-compliant if they have taken less than 80% of the capsules, as determined by either the capsules remaining or their checklist.

#### Physical activity

The subjects’ amount of physical activity is categorized as low, medium, or high. For example, someone with a sedentary lifestyle, who does only household jobs like cooking and sewing, and who works with a computer or in a laboratory does a low amount of physical activity. A medium level includes cleaning and childcare. A high level includes physical activities such as fast walking, cycling, swimming, and running [32].

#### Mental health

The GHQ-28 was developed by Goldberg in 1978 [28] and is used to identify psychiatric disorders in the general population. It is a 28-item measurement of mental health and emotional distress. The validity and reliability of the Persian version of the questionnaire have been established [33–36]. The subject is asked to compare their current psychological state versus their usual psychological state. It is suitable for assessing short-term psychiatric conditions rather than long-standing issues. The 28 items are categorized into four subscales: (1) somatic symptoms, (2) anxiety and sleep disorders, (3) social dysfunction, and (4) severe depression [37]. Each item is measured on a four-point scale (0–3).

#### Quality of life

The SF-36 questionnaire is a self-administered questionnaire on quality of life [38] with 36 items. The validity and reliability of the Persian version of the questionnaire have been established [39]. The SF-36 has been applied widely to estimate health-related quality of life changes in MS patients [40–42]. It evaluates the wellbeing of a patient from the patient’s viewpoint. The questionnaire has eight subscales: (1) physical functioning, (2) role limitations due to physical problems, (3) bodily pain, (4) perception of general health, (5) vitality, (6) social functioning, (7) role limitations due to emotional problems, and (8) mental health. It has two summary scores, one for the physical components and the other for the mental components.

#### Blood sampling and preparation

Serum levels of indices of inflammatory stress (high-sensitivity C-reactive protein or hs-CRP) and oxidative stress (malondialdehyde and total antioxidant capacity) will be measured. At the start and end of the study period, 12-hour-fasting blood samples (10 ml) will be collected from a forearm vein by a laboratory technician at Emam Reza Hospital Laboratory of Tabriz University of Medical Sciences. These samples will cooled to 4 °C and centrifuged for 15 min at 1000*g* (3000 rpm) within 30 min. Plasma samples will be divided in pyrogen-free tubes using pyrogen-free pipette tips and stored at −80 °C. Serum hs-CRP will be measured by an ELISA kit. The total antioxidant capacity of the plasma will be quantified by the ferric-reducing antioxidant power method. The malondialdehyde concentration will be measured by the thiobarbituric acid reactive substance method.

#### Food intake

Participants will record their dietary intake at three times during the study: before the intervention, after 2 months, and at the end. For each time point, their intake of food and beverages will be recorded for three nonconsecutive days (two weekdays and one day at the weekend) over 24 h [43]. Each type of food item and drink consumed will be entered into the software Nutritionist IV adapted for Iranian food as grams per day using the “Home Scales Guide.” The software will estimate the amount of energy, macronutrients and micronutrients consumed Their intake over the three days will be averaged for analysis and compared with the recommended dietary allowance.

### Data monitoring

All data will be stored in a database created for this trial by the Physical Medicine and Rehabilitation Research Center. The database will be in a secure room in the Physical Medicine and Rehabilitation Research Center to which only the principal investigator and a statistician will have access. The data will be entered into database by a trained technician. Each participant will have a sequential code number. Confidentiality of patient data will be maintained throughout the study. Blood samples will also be encoded and kept at −80 °C till analysis. The list of codes for each participant will be stored securely by the administrator of the Physical Medicine and Rehabilitation Research Center. Trial data will be accessible only to the investigator team and to the staff of the Physical Medicine and Rehabilitation Research Center. An independent data monitoring committee will be appointed according to the research integrity guidelines of the Physical Medicine and Rehabilitation Research Center. The data monitoring committee will meet every month to check the rate of recruitment, informed consent, adherence to the inclusion and exclusion criteria, compliance with the protocol, adverse outcomes, study progress, and the labeling and storage of blood samples. The data monitoring committee is independent from the sponsor.

### Adverse outcomes

All research staff will be asked to report any health problems related to the study at any time. All serious adverse events, whether associated or not with the intervention, will be documented on paper and electronic case report forms. All adverse events will be noted in the publication of the results.

### Statistical analysis

Data will be analyzed using the SPSS statistical software package version 16. Means ± standard deviation for quantitative data and frequency and percentage (%) for qualitative data will be reported. A *p* value of < 0.05 will be regarded as statistically significant. Baseline characteristics will be compared between the two groups by a chi-squared test or an exact test (Fisher’s exact test for 2 × 2 contingency tables or a Monte Carlo test for larger contingency tables) for categorical variables and independent *t*-tests for continuous variables. For all continuous data, a Shapiro–Wilk test and Levene’s test will be used to evaluate normality and the homogeneity of variances, respectively. Mean response scales measured over the 4-month study period will be analyzed with a mixed effects model.

### Ethical considerations

1. All participants will give informed consent before randomization and after the objectives and methodology of the trial have been explained to them.
2. Participants have the right to withdraw from the trial at any time without giving a reason, and will not be disadvantaged in any way for doing so.
3. Investigators are responsible for ensuring that all participant data remains confidential. All follow-up visits will occur in private.
4. Throughout the trial, the researchers will adhere to the Ministry of Health’s medical ethics statement.
5. This study has been approved by the ethics committee of the Research Vice-Chancellor of Tabriz University of Medical Sciences (IR.TBZMED.REC.1396.59) and it has been registered on the World Health Organization’s website for clinical trials (IRCT20161022030424N1).

## Discussion

None of the disease-modifying therapies presently being used for the treatment of MS are 100% efficacious. Furthermore, side effects related to the administration of these disease-modifying therapies have restricted the feasibility of combination therapy. Accordingly, there is a need for safe immunomodulatory medications to manage MS.

To the best of our knowledge, this is the first randomized clinical trial to evaluate the effect of probiotic SB on mental health, quality of life, and indices of inflammatory and oxidative stress in MS patients. No previous studies have targeted SB as a treatment for MS.

The commensal microbiota has an essential role in autoimmunity. It may be involved in autoimmune disorders including rheumatoid arthritis and MS [12, 44]. The growth in the incidence of MS and other immune-mediated disorders in western communities in recent decades may be linked to increased hygiene [45]. The hygiene hypothesis suggests that because of unnecessary hygiene, children are exposed to fewer antigens from microorganisms, causing their immune systems to become overactive and more likely to misidentify their tissues as foreign, resulting in immune-mediated diseases such as MS. Furthermore, some risk factors for MS have been associated with dysbiosis, for example obesity and vitamin D levels [46–48]. Probiotics may help to modulate the intestinal microbiota.

SB is a nonpathogenic probiotic yeast isolated from the peel of fruits such as lychees that grow in Indochina. It has anti-microbial, enzymatic, metabolic, and anti-toxin effects plus trophic activity [49]. Whilst the detailed mechanism by which SB may alleviate MS is not known, various potential mechanisms have been suggested. It enhances the effects of enzymes in the intestinal mucosa and through this improves the production of secretory IgA and the release of disaccharide enzymes, and contributes to the metabolism and absorption of carbohydrates [50]. The anti-inflammatory and immunomodulatory effects of probiotic SB have been found in previous studies [51–55]. Other mechanisms such as enhancing the tight junctions of gastrointestinal cells, neutralizing bacterial virulence, and improving the immune response of the gastrointestinal mucus have also been suggested [56]. SB can produce polyamines, which are important for cell growth and differentiation and can improve intestinal maturation [57].

Clinical trials have demonstrated the beneficial effects of SB for several disorders. Costanza et al. [58] found that if patients with heart failure take daily supplements of probiotic SB (1000 mg per day) for 3 months, there is a significant reduction in uric acid, hs-CRP and creatinine levels compared with those taking a placebo. Probiotic SB is well tolerated since there were been no reports of adverse events in study patients. In a double-blind randomized placebo-controlled trial, probiotic SB supplements for 12 weeks led to lower concentrations of a number of gut species, for example those of the Clostridiaceae family, which are related to systemic levels of bacterial translocation and inflammation indices [59]. It has been shown that for preterm neonates >30 weeks old, a twice daily dose of 50 mg/kg of probiotic SB enhances weight gain and feeding tolerance with no adverse effects.

However, there are some limitations that must be considered. First, eating probiotic-based foods is more appropriate and practical than taking supplements. Second, only the short-term effect of the intervention will be investigated because patients will be treated for only 4 months. Third, the levels of inflammatory and oxidative stress factors will be measured at only two time points throughout the study (at the baseline and at 4 months). Measuring the serum biomarkers every 2 months would give a clearer picture of the changes in the indices during this period. Fourth, fecal bacterial loads will not be examined either before or after probiotic consumption.

### Trial status

Planning for this study started in 2017. The first patient was randomized on 9 July 2018. Enrollment into the trial is ongoing. The populated SPIRIT checklist is provided as Additional file 1.

## Additional file

Additional file 1: SPIRIT Table. SPIRIT checklist of the protocol study. (DOC 128 kb)

## Data Availability

The datasets generated during the current study are available from the corresponding author on reasonable request.

## Abbreviations

CFU: Colony-forming units
GHQ-28: 28-item General Health Questionnaire
hs-CRP: High-sensitivity C-reactive protein
MS: Multiple sclerosis
SB: *Saccharomyces boulardii*
SF-36: 36-item Short Form questionnaire
